# Assessment of management approaches for hyperemesis gravidarum and nausea and vomiting of pregnancy: a retrospective questionnaire analysis

**DOI:** 10.1186/s12884-022-04922-6

**Published:** 2022-08-01

**Authors:** Rachel Mares, Adelene Morrow, Haley Shumway, Isain Zapata, David Forstein, Benjamin Brooks

**Affiliations:** 1 Department of Biomedical Sciences, Rocky Vista University College of Osteopathic Medicine, 255 E. Center St, Ivins, UT 84738 USA; 2 Department of Obstetrics, Central Michigan University, Mount Pleasant, MI 48859 USA; 3 Department of Biomedical Sciences, Rocky Vista University College of Osteopathic Medicine, Parker, CO 80134 USA; 4 Rocky Vista University College of Osteopathic Medicine, Office of the President, Parker, CO 80134 USA

**Keywords:** Nausea and vomiting of pregnancy, Hyperemesis Gravidarum, Nausea, Pregnancy, Vomiting, Morning sickness, Quality of life

## Abstract

**Background:**

Hyperemesis gravidarum is the most severe form of nausea and vomiting of pregnancy, or morning sickness. 2% of pregnancies in the United States are affected by hyperemesis gravidarum. The condition is characterized by severe vomiting in pregnant people, especially during the first trimester, often leading to hypovolemia and weight loss. The standard of care for hyperemesis and nausea and vomiting of pregnancy is commonly ineffective. We hypothesize that based on patient experience; the current treatment guidelines for hyperemesis are not clinically effective. Our objective was to identify the efficacy of the various management approaches that are currently in place for hyperemesis and nausea and vomiting of pregnancy.

**Methods:**

A questionnaire was designed based on diagnostic criteria, standard demographic identifiers, and common medications for the treatment of hyperemesis gravidarum. This questionnaire was distributed online to through hyperemesis and nausea and vomiting of pregnancy support groups, personal social media, and institutional email.

**Results:**

In our study, most participants diagnosed with hyperemesis gravidarum trialed at least three medications, most of which were ineffective and/or had severe side effects. The most used medication for treatment of hyperemesis gravidarum is ondansetron, a standard antiemetic, with fatigue and constipation being the most reported side effects. All data in the dataset was coded as categorical and analyzed using contingency tables using Mantel-Haenszel Chi square tests.

**Conclusions:**

The data presented in this research provides insight into the suffering that patients with these diagnoses face day-to-day due to the lack of efficacious, well-tolerated treatment options. Establishing this gap in treatment can facilitate the development of effective treatments that will provide relief for thousands of patients.

**Supplementary Information:**

The online version contains supplementary material available at 10.1186/s12884-022-04922-6.

## Introduction

Nausea and vomiting of pregnancy (NVP) are extremely common, taking place in 70-80% of pregnancies [1]. However, 0.3–3.6% of pregnant patients experience the most severe form of NVP known as Hyperemesis Gravidarum (HG), with debilitating symptoms [1]. A recently developed consensus definition for hyperemesis gravidarum, also referred to as the Windsor Definition, includes start of symptoms in early pregnancy (before 16 weeks gestational age); nausea and vomiting, at least one of which severe; inability to eat and/or drink normally; strongly limits daily living activities [2]. HG is further characterized by intractable vomiting, especially during the first trimester that is difficult to treat and often leads to hypovolemia and weight loss. HG is the single largest cause of early pregnancy hospitalization in the United States [3]. Patients with HG are commonly hospitalized due to weight loss that exceeds 5% of pre-pregnancy weight, dehydration, electrolyte imbalance, arrhythmias, and acid-base balance disturbance. HG can also be a driving factor in the development of metabolic disorders including acute kidney injury [4]. Despite the prevalence and significant morbidity associated with HG, high-quality research is still lacking in its basic etiology, treatment, and prevention. Most studies are limited by too few participants, biases, and uncontrollable confounding variables [5]. Furthermore, stigma, inappropriate management, and lack of investment have all played a role in impeding care for these patients [6].

The James Lind Alliance Priority Setting Partnership on HG determined that finding effective clinical management is the second highest priority in HG research [7]. Most research specific to therapeutic and pharmacologic interventions for HG does not include randomized control studies, and cross-study comparison is made challenging by inconsistent diagnostic criteria [2]. The etiology of NVP/HG still evades researchers and practitioners. Therefore, our current treatments are aimed at treating symptoms with general antiemetic regimens rather than at specific mechanisms in the pregnant patient. It is commonly accepted that NVP is multifactorial, potentially including hormone imbalances, placental disorders, and genetic causes [2]. Though there is no strong evidence to support serotonin, dopamine, or histamine being the direct causes of this condition, these are the targets for the standard antiemetic medications. Available therapies have remained virtually unchanged over the past few decades, and affected patients only respond partially to the antiemetics that are currently recommended for HG treatment [2].

Many HG patients have experienced sub-optimal management of their condition, specifically due to a lack of support from their healthcare providers [8, 9]. A study on a large cohort of affected patients found that those who reported that their provider was either not attentive to or not aware of the severity of their condition were more likely to also report psychiatric sequelae such as feelings of anxiety and depression [10]. Such approaches by providers may decrease the likelihood that patients seek care in a timely manner [11]. Although providers face a challenging situation due to the scarcity and low quality of studies evaluating the efficacy of antiemetics for HG [8]. The current lack of clinical attention, misinterpretation of symptoms, and delayed diagnosis and treatment of HG patients underlines a need to improve clinical care [12]. In a study involving an online survey of 249 patients with severe NVP or a formal diagnosis of HG, one in four were denied medications such as doxylamine to treat their condition [13]. Even when those with HG are treated with medications, the quality of evidence to support their use is slim. In a network meta-analysis and trial sequential analysis of randomized clinical trials of current drugs used to treat NVP, only ginger root had a moderate quality of evidence to support its use, and all other interventions had little evidence of efficacy [14].

Clinical pharmacotherapy guidelines for both NVP and HG involve a significant amount of trial and error with multiple combinations of antiemetics, vitamins, and supplements all while the patient is suffering [15–17]. Amalgamations of medications including doxylamine, dopamine antagonists, antihistamines, serotonin antagonists, phenothiazine medications, and vitamins/supplements are routinely used both in inpatient and outpatient settings to control symptoms of HG [16]. While the safety of these medications for the developing fetus is generally well established, they can cause unpleasant side effects for the pregnant person and these must be balanced against their efficacy at controlling symptoms [15, 17]. Neither the American College of Obstetricians and Gynecologists’ (ACOG) or the Royal College of Obstetricians and Gynecologists’ (RCOG) guidelines regarding NVP/HG report research that supports the efficacy of combination therapies using the medications mentioned above. In fact, much of the available research on these medications are based on their use as monotherapies, making treatment with combination therapies based solely on physician experience and preference [15, 18].

Our objective is to identify the subjective efficacy of the various management approaches that are currently in place for nausea and vomiting of pregnancy and hyperemesis gravidarum. The data presented in this research will provide insight into the suffering that patients with these diagnoses face day-to-day.

## Methods

### Data acquisition and study population

Before proceeding with the study, respondents were required to view and acknowledge written informed consent and were assured confidentiality. The questionnaire was administered via Google forms and each participant was asked to fill out one survey per child.

This questionnaire was distributed via institutional email, social media (including online international HG support groups and personal, non-HG related social media pages), as well as the Hyperemesis Education and Research (HER) Foundation website. Participants were incentivized with a raffle for gift cards of various amounts. Identifying information (email addresses) were collected at the time of data collection for the purpose of associating one participant with multiple pregnancies, resulting in multiple entries. This was necessary to separate those who may have had more than one child to determine the exact the number of participants, and sparse out duplicate data (if applicable). Identifiers were removed during data analysis and all information was kept on encrypted files on a password protected account and never shared with anyone outside of the research team. Inclusion criteria for this research included access to the internet, being assigned female sex at birth and birthing a live child. There was no limit regarding the number of pregnancies, how recently the pregnancy occurred, or country of residency.

### Questionnaire development

The questionnaire (Additional file 1 Appendix A) was created by adapting the diagnostic criteria used by the HELP Score Assessment developed by the HER Foundation [19]. Participants were asked to answer 26 questions, consisting of multiple-choice, Likert scales, and free-response question formats. The study was approved by the Rocky Vista University Institutional Review Board. A patient representative was consulted in the creation of the questionnaire to ensure accuracy and inclusivity from a patient’s perspective.

### Statistical analysis

All data in the dataset was coded as categorical and was therefore analyzed using contingency tables. The dimensions of these tables were dependent on the number of levels for each category. Since counts in cells were small in some cases, we base our main testing on Mantel-Haenszel Chi square tests for being more robust using smaller samples than asymptotic Pearson’s chi square tests. All data analyzes were performed using SAS/STAT v9.4 (SAS Institute Inc. Cary NC). Significance was declared at 95% confidence (*P*-value ≤0.05).

## Results

**Fig. 1:**
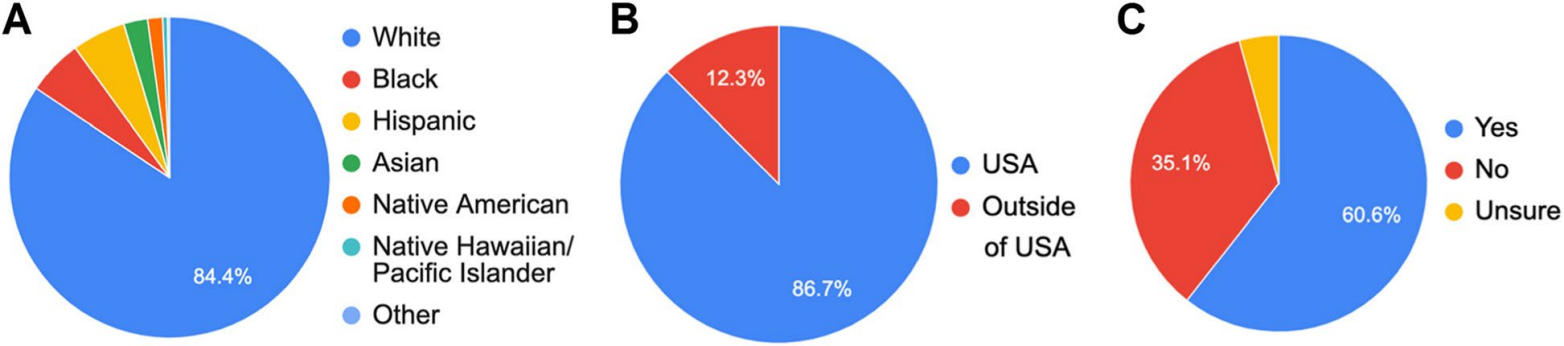
Respondent demographics. **A** Ethnicity and race of respondents. **B** Participants residing in the USA vs. outside the USA. **C** Have you even been diagnosed by a healthcare provided with Hyperemesis Gravidarum

A total of 786 participants responded to the survey and 1002 pregnancies were analyzed. Voluntary respondents completed a survey for each living biological child. As well as gathering demographic data, the survey assessed participants for symptoms congruent with HG /NVP**,** medication usage and side effects. The large majority of responses came from White Americans (Figs. 1A & B). Our data shows 60.6% of respondents were formally diagnosed with HG, with 4.3% reporting that they were unsure if they had been diagnosed (Fig. 1C).

### Medication usage

**Fig. 2:**
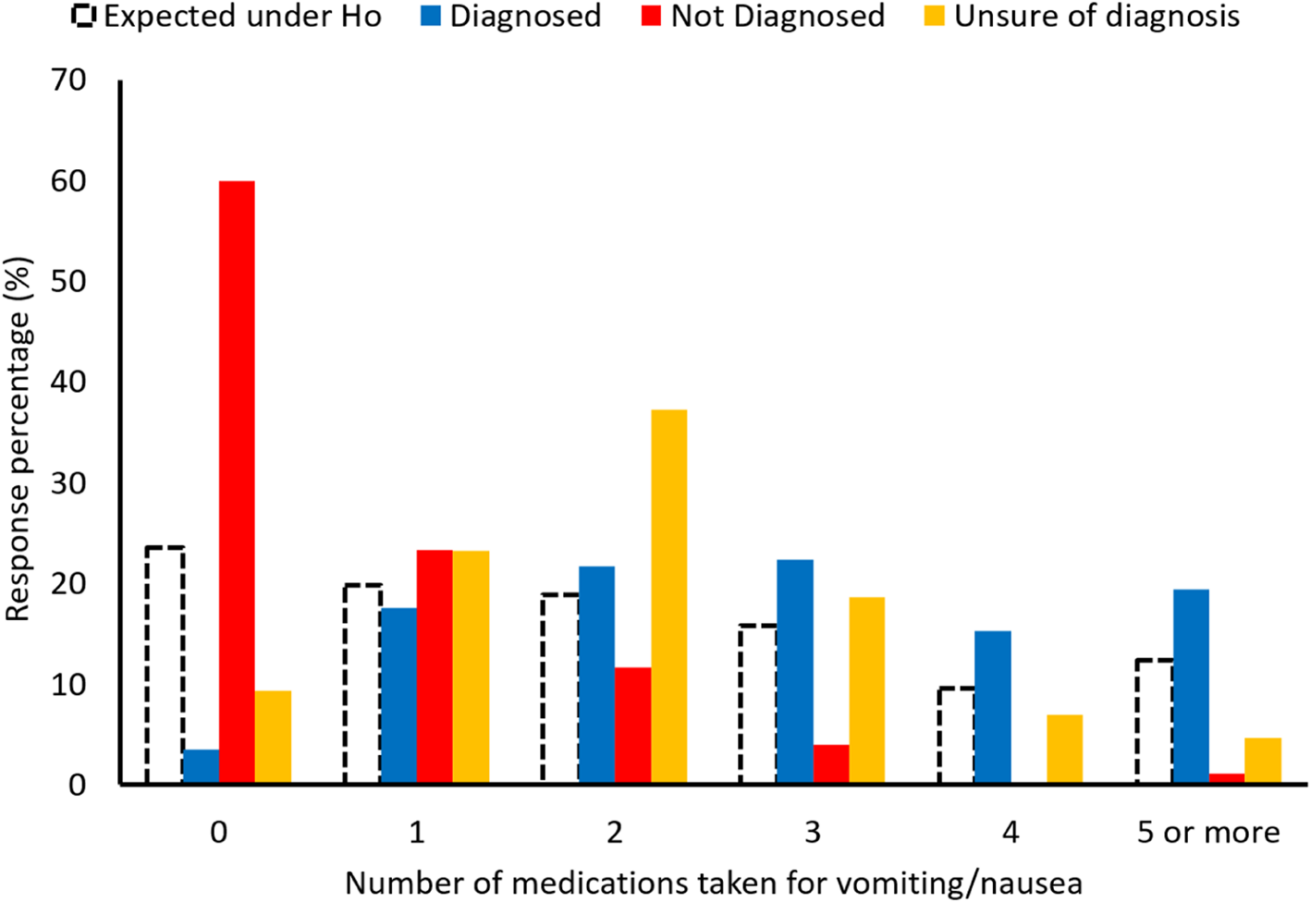
Number of medications women took depending on their diagnosis status. The expectation under Ho is the response percentage under a perfect allocation by group that would assume no effect; these expected values are calculated from a contingency table of the data. The larger the observed bars deviate from the expectation, the larger the effect in that group

**Fig. 3:**
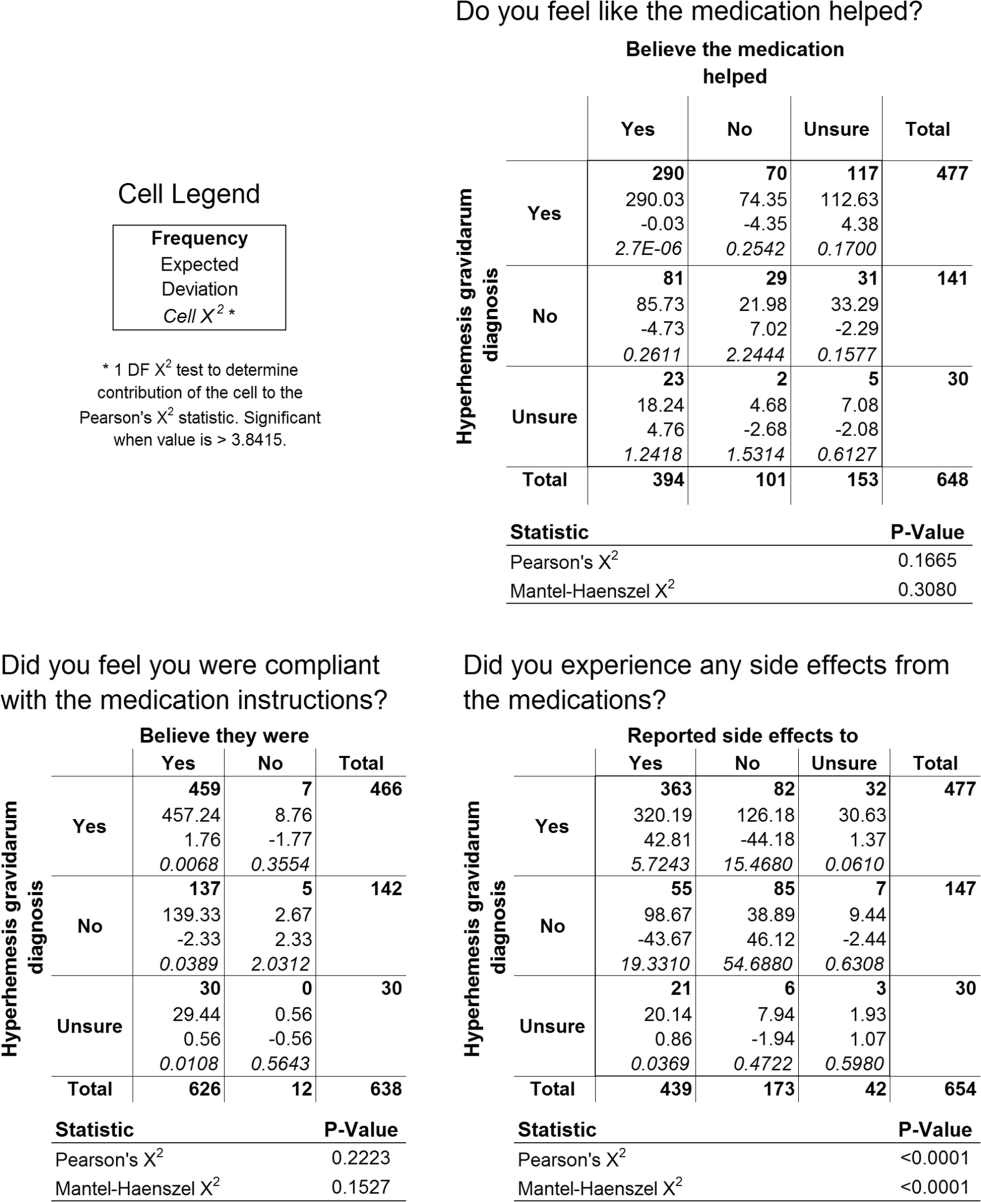
Three key questions from the survey were evaluated using contingency tables where pairwise associations were determined using Mantel-Haenszel Chi-square tests

**Fig. 4:**
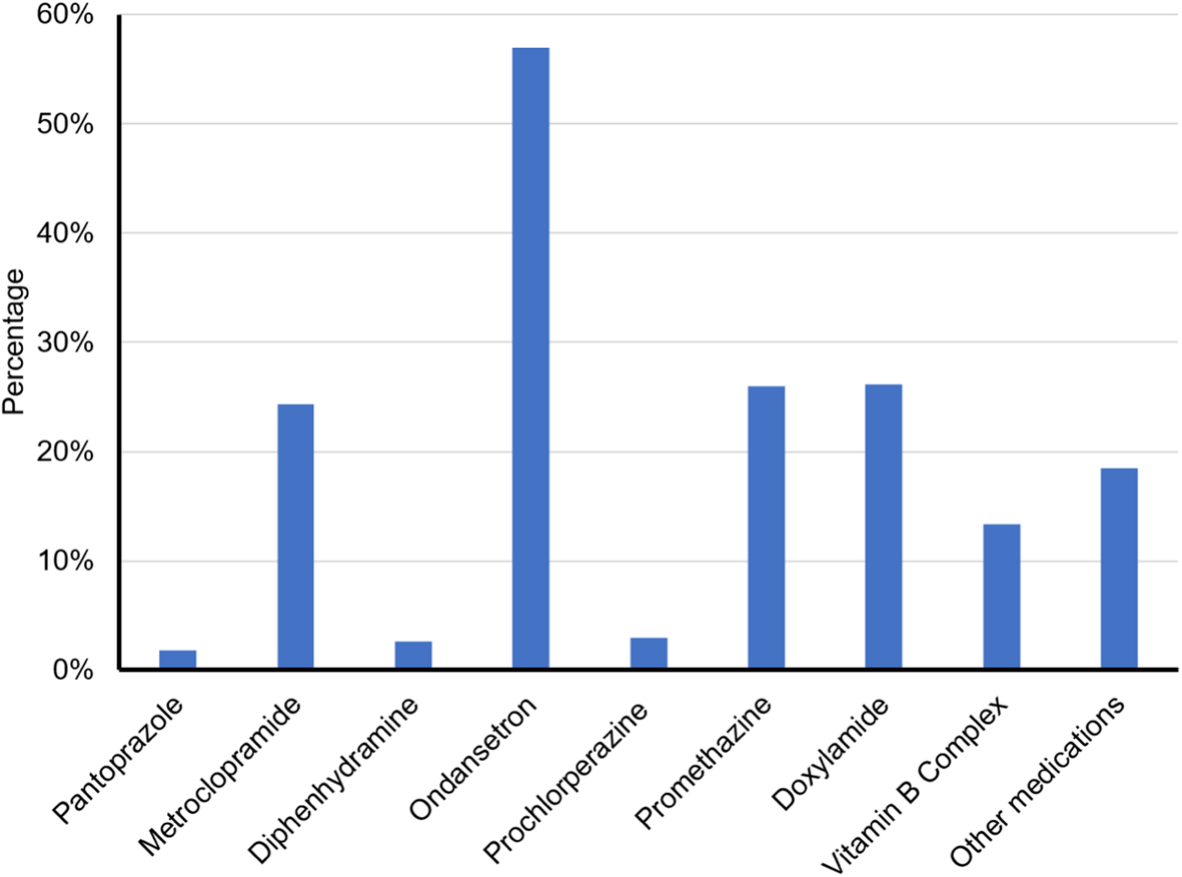
Commonly used medications frequency among surveyed women users formally diagnosed with HG

Of those diagnosed with HG, 22.4% took three medications, 15.3% took four, and 19.4% took five or more (Fig. 2). No associations were found between HG diagnosis and subjective, self-reported medication compliance (Fig. 3). The most used medication by patients with HG was ondansetron. Other commonly used medications were pantoprazole, metoclopramide, diphenhydramine, prochlorperazine, promethazine, doxylamine, and vitamin B complex (Fig. 4).

### Medication side effects

Sixty eight percent of patients who took medication for HG experienced side effects. These side effects included fatigue, constipation, and headache, and for some, even included anxiety and depression.

## Discussion

This study was conducted to elucidate gaps in the current management of NVP/HG. This information, including individual pharmacotherapy data, can help us identify risk factors for HG and its widespread effects on patients’ lives, which can be helpful for screenings, providing resources to patients, and developing further treatment options.

As such, our data shows that over one-half (57.1%) of respondents diagnosed with HG have taken at least three different medications to alleviate their symptoms, some relying on more than five separate daily medications. Furthermore, over two-thirds (68%) of these patients experienced side effects from their treatment options, ranging from fatigue and constipation to depression and anxiety, for some, though this is not necessarily associated with the number of medications prescribed. Our data suggests that we do not have an effective, well-tolerated treatment option for our NVP/HG patients. The Hyperemesis Education and Research (HER) Foundation has one of the most robust resources for management protocol of this disease, though it is often overlooked in clinical practice [20]. ACOG also has a detailed management protocol for NVP/HG, which, albeit very similar to the HER foundation’s, is more widely accepted amongst the Obstetrics community [15]. However, even the evidence presented in the Practice Bulletin used to create the guidelines is limited.

Pyridoxine plus doxylamine is first-line pharmacologic therapy for NVP and HG and is shown to be safe and effective [15–17]. However, around one-third of respondents taking this medication, reported central nervous system adverse effects (sleepiness/drowsiness) [15]. Another commonly used class of medications, dopamine antagonists, may lead to maternal extrapyramidal symptoms such as tardive dyskinesia, especially when combined with phenothiazine medications [15, 16]. Furthermore, both serotonin antagonists and phenothiazine derivatives may cause cardiotoxicity due to inhibition of cardiac potassium efflux channels potentially leading to QT prolongation [16, 17, 21, 22]. In brief, there are options for treatment, albeit these treatments have associated risks, therefore, a risk/benefit discussion with the patient regarding these treatments is imperative.

Most of these medications have at least proven to be more effective than placebo when treating NVP/HG [14, 17]. Although, ACOG Practice Bulletin #189 states that “no single approach has been proved to be more effective than the other” [15].

HG has far-reaching implications that outlast the physical illness. In a cross-sectional population-based study conducted in Norway, respondents suffering from more severe symptoms were found to have a physical quality of life close to that among patients with breast cancer, and a mental quality of life comparable to that seen among mothers with postpartum depression [23]. In an online survey of 377 patients who experienced HG, 18% met the criteria for posttraumatic stress syndrome [24]. In a study of over 5000 patients with HG, over half (52%) of participants had contemplated terminating a pregnancy due to the severity of their HG symptoms [25]. Two of five (40%) of participants in a separate Norwegian study expressed that they had contemplated abortion, and [8] subjects had a minimum of one elective abortion because of HG [8]. 19% of subjects switched their doctor because they were not receiving satisfactory HG treatment. These patients also expressed confusion about their prescribed pharmacological regimens, and 87.9% used one or more complementary and alternative medicines, but only 12.8% of whom reported any positive effect on HG symptoms. More than half of the interviewees believed their general practitioner had no knowledge of HG [8].

The limitations of this study include the retrospective and subjective nature of our data collection. We relied on the mothers’ memories of pregnancies that may have happened decades in the past. Furthermore, we had did not have the resources to verify the claims of diagnoses of Hyperemesis Gravidarum or Nausea and Vomiting in Pregnancy or the treatments of said diagnoses. We cannot rule out a self-selection bias due to respondents who have been severely affected with NVP/HG potentially being more willing to participate, therefore leading to the potential overestimation of severity. The use of the internet to disseminate the questionnaire may also isolate a population who do not have access to the internet or have low internet-literacy, though web-based recruitment has been shown to be valid in terms of study design [26].

## Conclusions

Despite the grave ramifications of this condition, many healthcare providers do not meet the needs of their HG patients. This is evident not only from the sheer number of medications that patients are trialing as shown in our data, but also from several studies regarding lessened quality of life and even post-traumatic stress disorder (PTSD). Furthermore, we discovered that more than half of the patients who are prescribed medication for their symptoms are experiencing a plethora of side effects. It is vital to reverse this trend; health care providers need to be aware of the high burden NVP/HG and its current treatment places on these patients.

Support and high-quality care are essential for this vulnerable population. However, the lack of a unified standard of care for NVP/HG patients prevents the condition from being widely recognized and downplays the need for effective interventions, which is considered one of the highest priorities of experts in this field regarding this condition. Without fully understanding the etiology of this multifactorial condition, the obstetrics community is at a loss for solutions to the root-cause of this problem. By emphasizing the lack of efficacious, well-tolerated treatment, we hope that this research will inspire the obstetrics community to better understand NVP/HG, including treatments and outcomes. Though pregnant people are a controversial study population, high-quality research is necessary to bridge this gap. Effective therapeutics for NVP/HG will give patients a better outlook on pregnancy, an improved relationship with their healthcare providers, and a higher quality of life during and after pregnancy.

## Supplementary Information

**Additional file 1.**

## Data Availability

The datasets used and/or analyzed during the current study are not made public because of privacy concerns but can be made available from the corresponding author on reasonable request.

## Abbreviations

HG: Hyperemesis Gravidarum
NVP: Nausea and Vomiting of Pregnancy
ACOG: American College of Obstetricians and Gynecologists
RCOG: Royal College of Obstetricians and Gynecologists
PTSD: Post-Traumatic Stress Disorder
HER: Hyperemesis Education and Research Foundation
