# Supplementary material for: Assessment of management approaches for hyperemesis gravidarum and nausea and vomiting of pregnancy: a retrospective questionnaire analysis
Source: BMC Pregnancy Childbirth. 2022 Aug 1;22:609. doi: 10.1186/s12884-022-04922-6 (PMC9341047; doi:10.1186/s12884-022-04922-6)
Supplement: Supplementary file 1 — Additional file 1. [file 12884_2022_4922_MOESM1_ESM.pdf]

Nausea and Vomiting of Pregnancy Survey

Informed Consent For Confidential Survey

You are invited to participate in a research study titled: Socioeconomic and Other Influences in Nausea and Vomiting of Pregnancy and Hyperemesis Gravidarum.

This study is being conducted by medical students, Rachel McCann and Haley Shumway, under the direction of Ben Brooks, PhD, Assistant Director of the MSBS Program and Amanda Brooks, PhD, Director of Research and Scholarly Activity and Associate Professor of Molecular Biology at Rocky Vista University.

The purpose of this study is to investigate Hyperemesis Gravidarum and nausea/vomiting during pregnancy or “morning sickness” outcomes with consideration for the socioeconomic influences, race, age, support system, and pregnancy history. Dependent on your answers, we may ask for your help in upcoming studies targeting a gene that may be indicated in morning sickness. The results will be used to further understand what factors influence morning sickness and it’s outcomes.

Participation in this study is entirely voluntary at all times. You can choose not to participate at all or to leave the study at any point. If you decide not to participate, or to leave the study, there will be no penalty or loss of benefits to which you are entitled, or any effect on your relationship with the researcher(s), or any other negative consequences.

You are being asked to take part in this study because you are a mother who has been personally affected by morning sickness or know someone who has.

If you agree to participate, you will be asked to fill out one survey per living biological child about your pregnancy history along with symptoms as well as other factors such as socioeconomics, race, etc. This survey should take around 5 minutes to complete.

The survey will be collected via google forms. All of your responses to this survey will remain confidential by the investigators and will not be linked to you once de-identified. Only your email will be collected during the study. Dependent on your answers, we may contact you to participate in the next step of this research. You are free to withdraw from this study at any time. However, once you submit your completed survey, there will be no way to withdraw your responses from the study because the survey will be kept confidential.

Study data will be kept in digital format. This information/data will be stored and safeguarded in a restricted access folder on a password protected computer, and your survey responses will be identified only by the investigators listed above.

Any risks to you associated with this study are not expected to be greater than anything you encounter in everyday life. While you may not experience any direct benefits from

participation, information collected in this study may benefit others in the future by helping to identify the various factors that contribute to morning sickness. More broadly, this research will help physicians and researchers continue to understand these diagnoses and how to better predict and treat them.

If you have any questions regarding the survey or this research project in general, please contact the principal investigator, Haley Shumway, at haley.shumway@rvu.edu or her faculty mentor, Benjamin Brooks at bbrooks@rvu.edu.

If you have any questions about your rights as a research participant, please contact the Rocky Vista University IRB Compliance Administrator at 720-874-2481 or ldement@rvu.edu.

By completing and submitting this survey, you are indicating your consent to participate in this study.

[redacted] Switch account

* Required

Email *

Your email

I give my informed consent to take this survey. *

- ◯ Yes
- ◯ No

Ethnicity/race *

- [ ] White
- [ ] Black or African American
- [ ] Hispanic
- [ ] Asian
- [ ] Native American
- [ ] Native Hawaiian or other Pacific Islander
- [ ] Other:

How old were you at the time of this pregnancy? *

- ○ 17 or younger
- ○ 18-20
- ○ 21-25
- ○ 26-29
- ○ 30-35
- ○ 36-39
- ○ 40 or older

What is the highest level of school you had completed or the highest degree you had received at the time of this pregnancy? *

- Less than high school degree
- High school degree or equivalent (GED)
- Some college but no degree
- Associate degree
- Bachelor degree
- Graduate degree

Household Income at the time of this pregnancy *

- $0 - $19,000
- $20,000- $49,000
- $50,000 - $99,000
- $100,000 - $149,000
- $150,000 - $199,000
- $200,000 - $249,000
- $250,000+

Which of the following categories best describes your employment status at the time of pregnancy? *

- Employed, working 1-39 hours per week
- Employed, working 40 or more hours per week
- Not employed
- Disabled
- Other:

Which demographic area did you reside in at the time of this pregnancy? *

- Northwest
- Southwest
- Midwest
- Southeast
- Northeast
- Outside of US

How many living biological children do you have? *

Your answer

For which pregnancy are you filling this survey out and what sex is your child? Example: First pregnancy with female child: "1, female". *

Your answer

Please rank your pregnancies in order of severity from worst to best in terms of "morning sickness". For example: If you have had 3 pregnancies and your morning sickness was worst with your third pregnancy, then your first, then your second; please respond in the following manner: "3>1>2". *

Your answer

Have you ever been diagnosed by a healthcare provider with Hyperemesis Gravidarum? *

- ○ Yes
- ○ No
- ○ Unsure

While pregnant please check if you experienced any of the following: *

- [ ] Weight loss (>5% of weight)
- [ ] Dehydration requiring IV fluids
- [ ] Hospitalization
- [ ] Electrolyte abnormalities
- [ ] Nausea
- [ ] Vomiting
- [ ] None of the above
- [ ] Other:

How much weight did you lose? *

- ○ 0-5 lbs
- ○ 5-10 lbs
- ○ 10-15 lbs
- ○ 15-20 lbs
- ○ 20-25 lbs
- ○ >25
- ○ Did not lose or gain any weight
- ○ N/A

How severe would you rate your nausea while pregnant for the majority of the time? *

	1	2	3	4	5	
No nausea	◯	◯	◯	◯	◯	Severe Nausea

How severe would you rate your vomiting while pregnant? *

	1	2	3	4	5	
No vomiting	◯	◯	◯	◯	◯	Vomiting >5x per day

Did you take any medications specifically for nausea or vomiting while pregnant? If you can remember the name of the medication you took please write it under other. *

- [ ] Yes
- [ ] No
- [ ] Unsure
- [ ] Other:

Number of medications taken for nausea/vomiting?

- 0
- 1
- 2
- 3
- 4
- 5+

Do you feel like the medication helped? *

- Yes
- No
- Maybe
- N/A
- Other:

Did you experience any side effects from the medications? *

- Yes (please describe your symptoms in the next question)
- No
- Unsure
- N/A

If you answered yes to the question above, please describe the side effects you experienced. *

Your answer

Do you feel you were compliant with the medication instructions? *

- Yes
- No
- Maybe
- N/A

How do you feel you emotionally coped with the nausea, vomiting, and retching?

- Normal
- Tired but mood was okay
- Slightly less than normal
- Tolerable but difficult
- Struggling: moody, emotional
- Poorly: irritable, depressed
- N/A

Are you aware of anyone else in your family who suffered from severe morning sickness? *

- Mother
- Grandmother
- Sister
- None

Average number of hours you were UNABLE to work adequately at your job and/or at home due to being sick: *

- 0
- 1-2 (slightly less hours)
- 3-4 (could work part time)
- 5-7 (could only do a little work)
- 8-10 (could not care for family)
- 11+ (could not care for yourself)

How many hours out of the day did you experience symptoms? *

	1	2	3	4	5	
1 hour	○	○	○	○	○	24 hours

How strong was your support system? *

	1	2	3	4	5	
Poor	○	○	○	○	○	Strongly supported

How long did your symptoms last during pregnancy? *

- ○ First trimester
- ○ Through second trimester
- ○ Entirety of pregnancy

Do you feel like your symptoms compromised your overall enjoyment of pregnancy? *

- ○ Yes
- ○ No

Submit

Clear form

Never submit passwords through Google Forms.

This content is neither created nor endorsed by Google. Report Abuse - Terms of Service - Privacy Policy

Google Forms
